# The Book World of Medicine and Science

**Published:** 1899-03-18

**Authors:** 


### The Book World of Medicine and Science.

Fry's Law of Vaccination. With Introduction, Notes, and Indexes. By A. F. Vulliamy. Seventh Edition. Pp. 180. (London: Knight and Co. 1899. Price 7s. 6d.)

Fry's "Law of Vaccination" has, in this edition, been brought down to include that concession to fatuity known as the Vaccination Act of 1898, and the orders of the Local Government Board relating thereto. It is prefaced by an admirable introduction—historical in method, terse and succinct in manner—which, of logical necessity, lays bare the imbecility of what the author rightly calls the great experiment. There is no need for us here to discuss the provisions of the new Act. Suffice it to say that in this book they are clearly set forth. The incidental comments by Mr. Vulliamy are eminently sane. We quote one pointed passage: "Upon vaccination officers mainly will depend the responsibility if any great outbreak of small-pox occurs through their neglect of the duties cast upon them by law, either through their own negligence or from a desire to stand well with guardians who disbelieve in vaccination." "No dread of boards of guardians should influence them; for the majority . . . will esteem an officer who kindly and courteously but efficiently carries out the duties imposed on him by law." The pity of it is that so few can properly appreciate, from experience, the horrors now threatening our country.

Photo-micrography. By Edmund J. Spitta, L.R.C.P., M.R.C.S., F.R.A.S. With 41 half-tone reproductions from original negatives and 63 text illustrations. (London: The Scientific Press, Limited, 28 and 29, Southampton Street, Strand. 1899.)

This book is one which reflects credit alike on its author and its publisher. Photo-micrography is now so largely utilised as an adjunct to scientific research that a clear exposition of the *modus operandi* will be welcome to many who wish to become acquainted with the technique necessary for the successful production of good negatives; and the amateur also will doubtless be glad to have the way cleared by a lucid exposition of the line of action to adopt as well as of the numerous errors to avoid. Dr. Spitta has usefully classified his directions under the heads of low, medium, and high-power work respectively; and in selecting this method he has made a wise choice, for anyone at all conversant with the practical details of photo-micrography is well aware of the widely different treatment to be adopted in the different cases. Medium work, with which the beginner should start, and which includes magnifications of from 50-500 diameters (Dr. Spitta, however, gives a range of 10-600 diameters, which we consider too wide), does not entail the difficulties of even illumination which stand in the way of very low or very high power work, nor is the very great trouble of focussing so acutely felt as when the magnification exceeds 900 or 1,000 diameters. Good directions are given as to the management of the limelight, but we think that the use of the arc is, perhaps, rather under-rated, especially for high-power work. Much of the difficulty in connection with its use is found to disappear if the carbons are obliquely cut, so as to lap over each other. The explanations given of the effects of various combinations of eye-pieces, with objectives of different angular aperture, are very much to the point, the more so as they are often overlooked by photographers in practice; this is especially the case with amateurs who have not mastered the intricacies of numerical aperture and other optical matters relating to the construction of lenses. A set of useful tables, together with a list of common defects in micro-photographs and their causes, conclude an admirable work which can be heartily recommended to all interested in this branch of microscopy or photography.

The Thermal Waters of Bath. By Gilbert A. Bannatyne, M.D. (J. Wright and Co., Bristol. Pp. 87. 1899. Price 2s.)

Dr. Bannatyne's book does not materially differ from any other that has been written by a fashionable physician about a fashionable watering place. It begins in the approved fashion with matter introductory and historical; in this case much about Bladud and Nash. It provides the usual tables clearly demonstrating the wonderfully low death-rate amongst the favoured residents, and the advantages of the local climate. Elaborate analyses—with many decimals—of the "thermal waters" are given. The taste of these particular waters is said to be "metallic"—Sam Weller said "like warm flat-irons." Mr. Smauker ascribed the flavour to the "Killybeate." Professor Attfield has found 1.2 grains of carbonate of iron in an imperial gallon. A full account of the baths is given—like the baths everywhere else—"the finest in Europe." The Bath waters, Dr. Bannatyne tells us, are found beneficial in rheumatism, gout, rheumatoid arthritis, sciatica, neuralgia, digestive disorders, anæmia, chlorosis, peripheral neuritis, hemiplegia, tabes, chorea, debility, respiratory disorders, diseases of the heart, and diseases of women. More than this could not be said of any pink pills, green globules, or patent cocoas. Nevertheless we have in Dr. Bannatyne's little book a good description of one of the most important of the English spas. Places like Bath, which have done much to render their therapeutic resources available for public use, ought to be rewarded by receiving public support.

The Secret of Good Health and Long Life. By Haydn Brown, L.R.C.P., M.R.C.S. Second edition. Sixth thousand. Pp. 156, long 8vo. (London: J. Bowden. 1898. Price 1s.)

Mr. Haydn Brown has written a really valuable little book of great incisiveness and good sense. It deals with its subject in a manner at once original and without bias; on certain points with refreshing candour. As the author says, it attempts to present principles of right living in a manner that will appeal to simple thought and common sense. It is the "little foxes" that spoil the grapes, and Mr. Brown rightly urges parents and guardians to give cognisance to "predisposing beams" rather than to "exciting motes." We hope they will take the advice, but if they do "our occupation's gone."

Whitaker's Naval and Military Directory and Indian Army List. 1889. (London: J. Whitaker and Sons. Price 5s.)

This edition is a considerable improvement upon last year's. The alphabetical list now contains the names of about 45,000 officers of the two services, including commissioned officers on the active list of Navy, Army, and Royal Marines, together with retired flag officers, captains, commanders, staff captains, &c., &c., of the navy and field officers of the army, giving particulars of their age, seniority, station or address, and war services. In addition to this list, which occupies about two-thirds of the book, there are an Army List, a Navy List, and a list of the Royal Indian Marine, a descriptive account of all medals and ribbons, and other matters of interest. A most useful directory to the services.

